# Controlled Trials in Children: Quantity, Methodological Quality and Descriptive Characteristics of Pediatric Controlled Trials Published 1948-2006

**DOI:** 10.1371/journal.pone.0013106

**Published:** 2010-09-30

**Authors:** Denise Thomson, Lisa Hartling, Eyal Cohen, Ben Vandermeer, Lisa Tjosvold, Terry P. Klassen

**Affiliations:** 1 Cochrane Child Health Field, Department of Pediatrics, University of Alberta, Edmonton, Canada; 2 Alberta Research Centre for Health Evidence, Department of Pediatrics, University of Alberta, Edmonton, Canada; 3 Hospital for Sick Children, Department of Pediatrics, University of Toronto, Toronto, Canada; Medical Research Council South Africa, South Africa

## Abstract

**Background:**

The objective of this study was to describe randomized controlled trials (RCTs) and controlled clinical trials (CCTs) in child health published between 1948 and 2006, in terms of quantity, methodological quality, and publication and trial characteristics. We used the Trials Register of the Cochrane Child Health Field for overall trends and a sample from this to explore trial characteristics in more detail.

**Methodology/Principal Findings:**

We extracted descriptive data on a random sample of 578 trials. Ninety-six percent of the trials were published in English; the percentage of child-only trials was 90.5%. The most frequent diagnostic categories were infectious diseases (13.2%), behavioural and psychiatric disorders (11.6%), neonatal critical care (11.4%), respiratory disorders (8.9%), non-critical neonatology (7.9%), and anaesthesia (6.5%). There were significantly fewer child-only studies (i.e., more mixed child and adult studies) over time (P = 0.0460). The proportion of RCTs to CCTs increased significantly over time (P<0.0001), as did the proportion of multicentre trials (P = 0.002). Significant increases over time were found in methodological quality (Jadad score) (P<0.0001), the proportion of double-blind studies (P<0.0001), and studies with adequate allocation concealment (P<0.0001). Additionally, we found an improvement in reporting over time: adequate description of withdrawals and losses to follow-up (P<0.0001), sample size calculations (P<0.0001), and intention-to-treat analysis (P<0.0001). However, many trials still do not describe their level of blinding, and allocation concealment was inadequately reported in the majority of studies across the entire time period. The proportion of studies with industry funding decreased slightly over time (P = 0.003), and these studies were more likely to report positive conclusions (P = 0.028).

**Conclusions/Significance:**

The quantity and quality of pediatric controlled trials has increased over time; however, much work remains to be done, particularly in improving methodological issues around conduct and reporting of trials.

## Introduction

Controlled trials have led to some notable advances in pediatric medicine. Famously, the trials of polio vaccines in many countries during the 1950s paved the way for the near-eradication of this disease [1], [2]. During the 1950s and 1960s, neonatologist William Silverman used the randomised controlled trial study design to test several commonly-used treatments, often demonstrating their lack of effectiveness[3]–[6]. More recently, clinical trials in childhood cancer have resulted in steady improvement in survival rates [7]. Overall, however, it has long been recognized in the pediatric research community that there is a paucity of child-relevant evidence available from controlled trials[8].

The reasons for the lack of controlled trials in child health are numerous, including among others: parental reluctance to agree to the participation of their children in research studies involving unproven treatments; the difficulty of recruiting adequate sample sizes, particularly for uncommon conditions; ethical concerns; and perceptions among drug manufacturers that testing drugs in children brings the risk of increased liability [9], [10].

The fact that many interventions carried out on children are inadequately tested has direct implications for child health. For example, the lack of evidence about the efficacy and effectiveness of many drugs means that selection and dosage of drugs administered to children is often done at the discretion of individual physicians, with choices based on extrapolation from studies in adults, a practice known as “off-label prescribing” [11]. Due to children's differing developmental and physiological processes, such extrapolation is often not appropriate. Adult-extrapolated dosing in children may either lead to overdosing (with the possibility of toxic effects) or under dosing (ineffective therapy) [11]. Moreover, outcomes in children, particularly young children, can be different than those in adults tested with the same intervention (see for example[12]); also, the goals of treatment for children with chronic or life-threatening conditions are often to ensure decades of life, with as high a quality of life as possible, whereas adults usually have to live with the effects of treatment for a much shorter time [13].

In recent years, there have been developments in both the U.S. and Europe that appear likely to increase the number of controlled trials in pediatrics. Legislation, such as the US Best Pharmaceuticals for Children Act (2002, renewed 2007) and Pediatric Research Equity Act (2003) and the European Pediatric Rule (2007), has been introduced encouraging drug manufacturers to evaluate the safety and effectiveness of products in pediatric patients, if the intervention is likely to be used in a substantial number of children, or provide a more meaningful therapeutic benefit to pediatric patients than existing treatments [10], [14]–[16]. For example, in the US, amendments in 1997 to Federal Food, Drug, and Cosmetic Act, along with the 2002 Best Pharmaceuticals for Children Act (BPCA), provided economic incentives to pharmaceutical companies to encourage them to perform research on the safety, efficacy, dosing and unique risks associated with medications for children [10] [17]. This legislation, of course, specifically addresses testing of pharmaceuticals. There is currently no equivalent requirement for testing of non-drug interventions, such as behavioural or social programmes; trials of these interventions make up a substantial proportion of child health trials, as will be discussed later in this paper.

Given the need for child-specific evidence for the appropriate delivery of health care interventions, a survey of the state of existing research is valuable, both as a benchmark and as a guide for future improvement. We used the Cochrane Child Health Field's Trials Register, a database of over 30,000 randomized controlled trials (RCTs) and controlled clinical trials (CCTs) in child health, to carry out this survey [18]. We defined RCTs and CCTs following the guidance in the Cochrane Handbook for Systematic Reviews of Interventions, whereby randomized controlled trials are conducted on groups established by random allocation which is explicitly described by the authors; the term “controlled clinical trial” is applied to studies for which it is not possible to determine if randomization was used, or if quasi-random methods of assignment were used [19]. The objective of our study was to describe child health RCTs and CCTs, published between 1948 and 2006, in terms of quantity, methodological quality, and publication and trial characteristics.

## Results

Our description of pediatric trials is based on analysis of the Child Health Field Trials Register as a whole, along with in-depth analysis of a random sample of trials from each time period.

### Characteristics of the entire Child Health Field Trials Register

**Figure 1:**
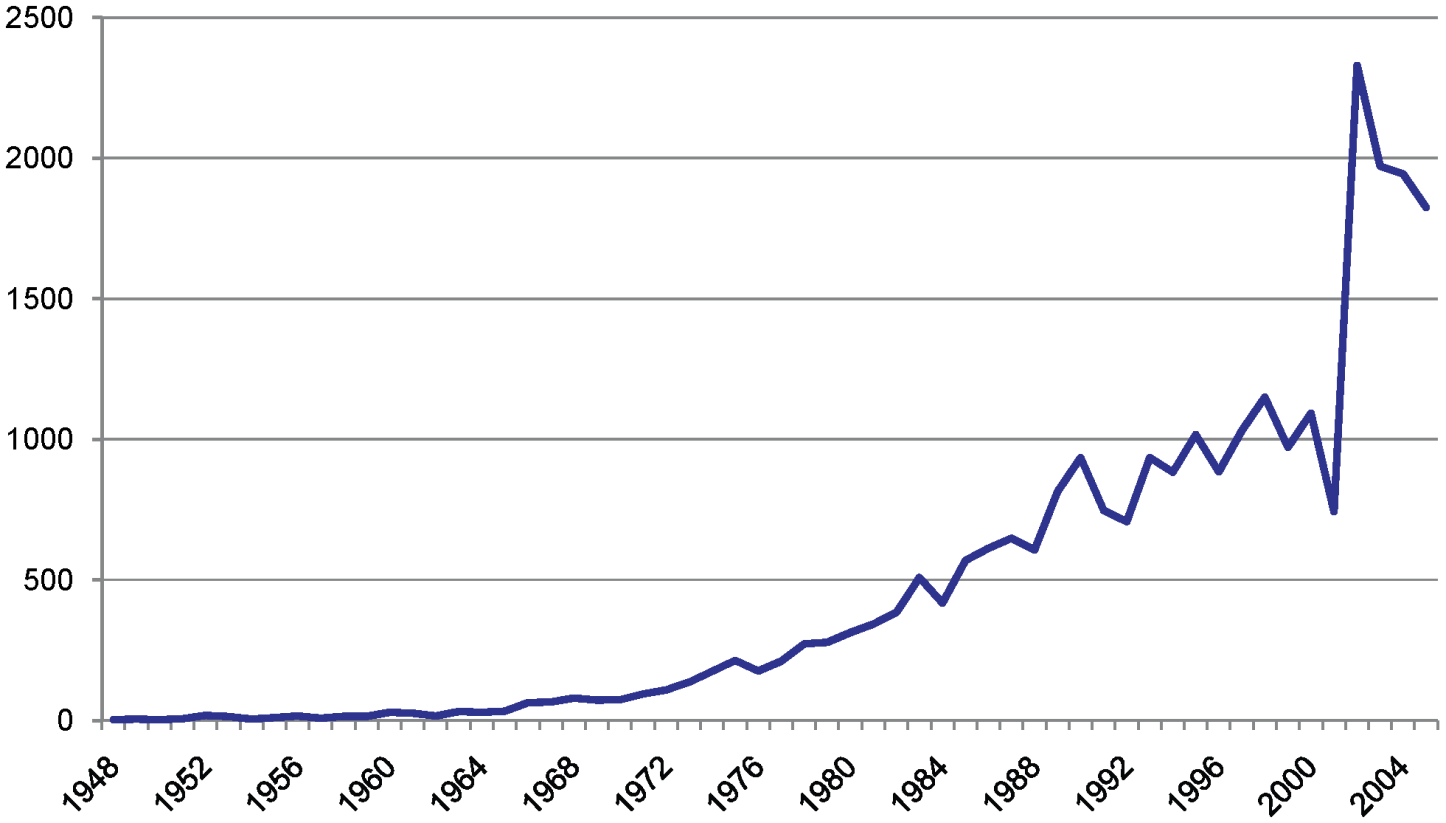
Number of RCTs and CCTs in the Child Health Field Trials Register, 1948–2005. NB. The numbers are only graphed to 2005 because this is the last complete year in the Trials Register. The dip in trials in 2001 is unexplained.

The number of trials per year in the Child Health Field Trials Register ranged from a low of 3 (1948) to a high of 2,722 (2004). The number of RCTs and CCTs published each year has, overall, steadily increased (Figure 1).

**Figure 2:**
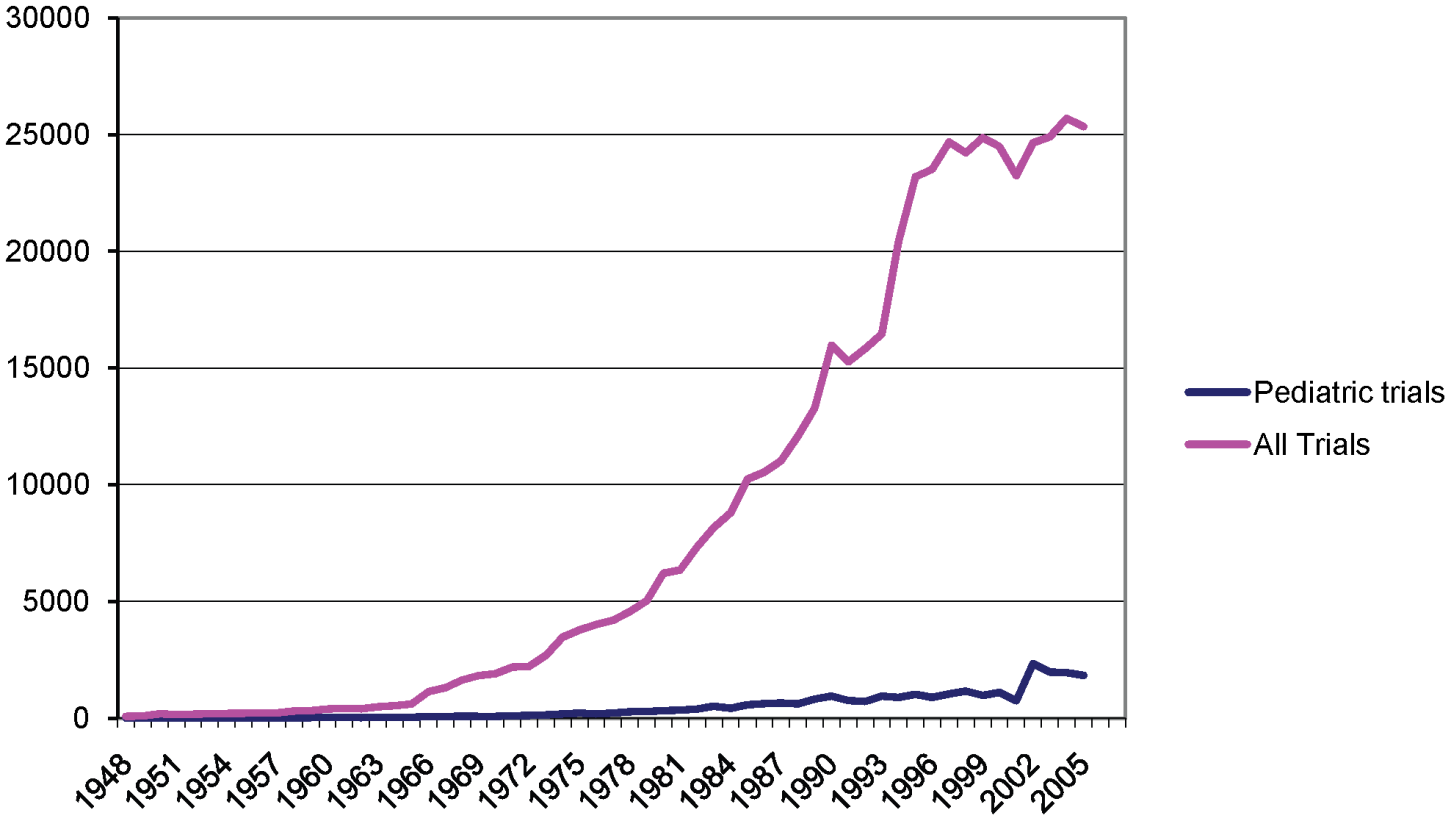
Trials in the Child Health Field Trials Register against trials in CENTRAL, 1948-2005. NB: In both the Trials Register and CENTRAL, there is a dip in numbers in 2001. This is unexplained.

We analysed pediatric trials as a proportion of total trials in the Cochrane Central Register of Controlled Trials (CENTRAL), a bibliographic database of controlled trials that have been identified through handsearching and database searching (Figure 2). This analysis was done in order to estimate the proportion of total published trials that are concerned with child health. We did this comparison for the years 1948 to 2005, since our Trials Register was not complete for 2006 at the time of this study. The number of trials in the Child Health Field Trials Register represents a proportion of all trials in CENTRAL ranging between 1.66% (1948) and 10.7% (2004) (Figure 2).

### Characteristics of pediatric trials, based on the analysis of our sample of 578 trials

Table 1 presents the characteristics of a sample of 578 studies from the Child Health Field Trials Register. This sample was taken from the Trials Register according to a protocol outlined in our Materials and Methods section, and data extraction was carried out to identify publication and trial characteristics for each study, along with assessment according to the Jadad scale[20].

**Table 1:** Characteristics of trials sampled from the Child Health Trials Register (n = 578).

Variable	Category	Estimate % (95% CI)
Design	CCT	10.9 (7.8, 13.9)
	RCT	89.1 (86.1, 92.2)
Intervention	Pharmaceutical	67.2 (61.2, 73.2)
	Non-Pharmaceutical	32.8 (26.8, 38.8)
Journal	Pediatric	32.8 (26.6, 39.0)
	Medical	61.5 (55.1, 67.9)
	Other	5.7 (2.4, 8.9)
Country of corresponding author	USA	38.3% (32.0%, 44.6%)
	UK	10.7% (6.6%, 14.9%)
Jadad Score	Median (IQR)	2 (1, 3)

Almost all (96.0%) of the trials were published in English. The corresponding authors for almost half (49.0%) of the trials were based in either the United States or the United Kingdom. The overall percentage of child-only trials was 90.5% (95% CI: 86.4, 94.5%). 396 (68.5%) trials investigated drug products and 173 (29.9%) were placebo-controlled

The most frequent diagnostic categories of the included trials were: infectious diseases (13.2%), behavioural and psychiatric disorders (11.6%), neonatal critical care (11.4%), respiratory disorders (8.9%), non-critical neonatology (7.9%), and anaesthesia (6.5%).

#### 1. Trends over time

There was a trend towards fewer child-only studies (i.e. more mixed child and adult studies) over time (P-value: 0.046); this value is statistically significant at the 5% level, but not under any correction for multiple testing.

**Figure 3:**
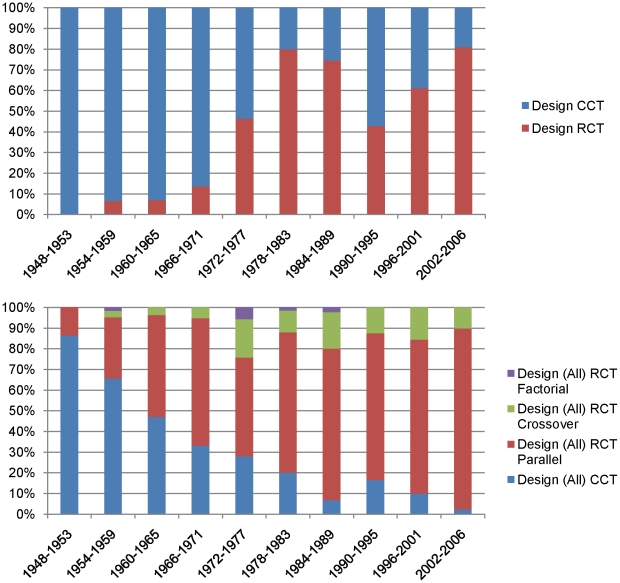
Characteristics of the study design in our sample. a. Relative proportions of RCTs and CCTs over time from 1948 to 2006. b. Designs of RCTs over time from 1948–2006.

The proportion of RCTs to CCTs increased significantly over time (Figure 3a) (P<0.0001). The majority of RCTs were of a parallel design (Figure 3b).

The proportion of multi-centre trials increased over time (P = 0.002). There was no significant change in the proportion of multinational studies over time, however the sample size of multinational trials in our sample was small (n = 17) (P = 0.15).

While the number of trials did increase over time, the median sample size of studies followed a parabolic trend—the numbers went steadily down until the late 1980s and have started to come up again since then (Table 2).

**Table 2:** Sample sizes over time.

	1948–1953	1954–1959	1960–1965	1966–1971	1972–1977	1978–1983	1984–1989	1990–1995	1996–2001	2002–2006
Mean	257	714	354	793	592	195	225	182	136	281
Median	101	100	96	70	54	50	40	50	60	99

#### 2. Methodological Quality

**Figure 4:**
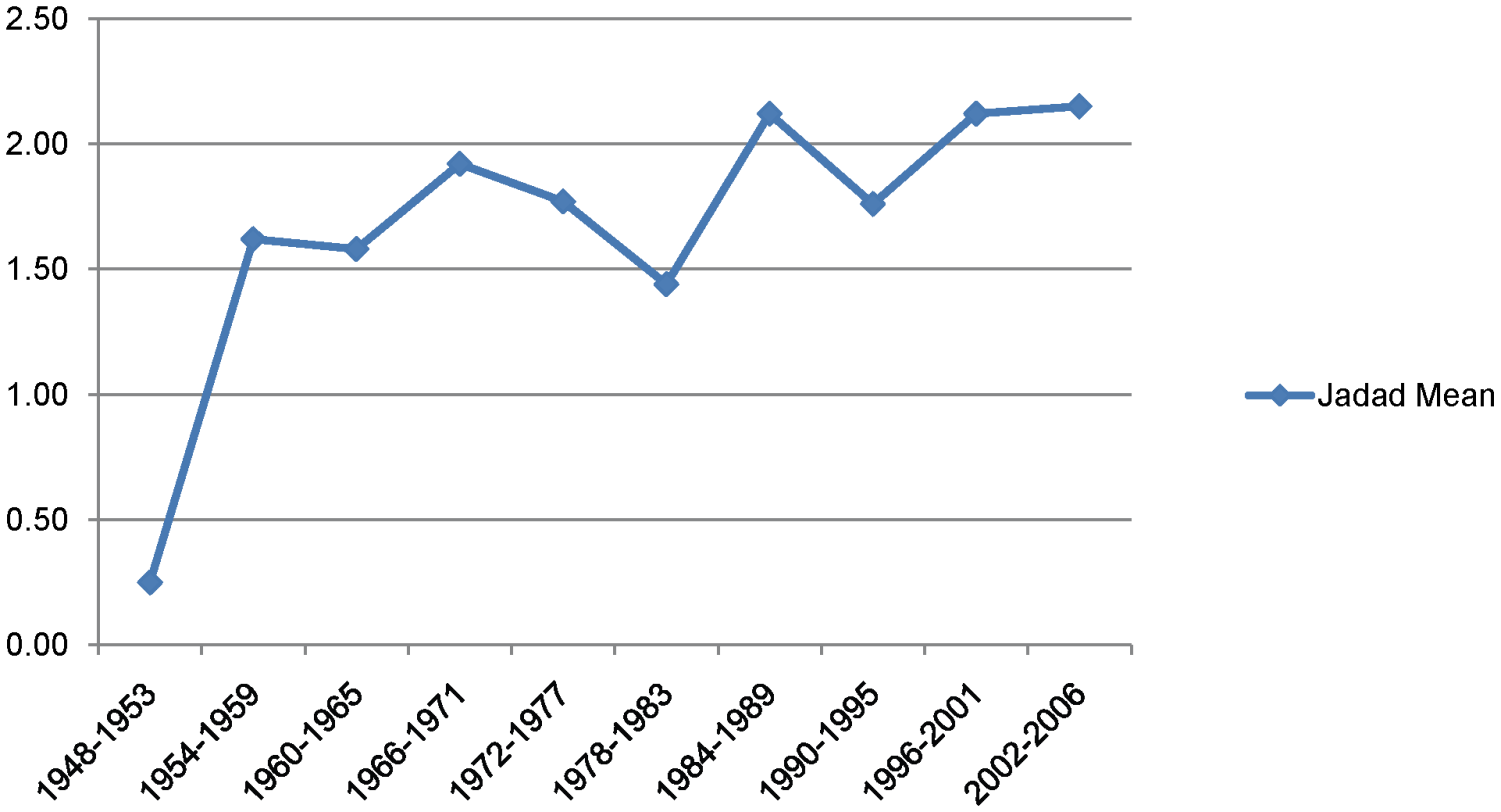
Mean Jadad score over time.

Methodological quality as assessed by the Jadad score increased significantly over time (P<0.0001); we also reanalyzed the data minus the first period (1948–1953) (where the average Jadad score was much lower) and the increase over time was still highly significant (p = 0.0002) (Figure 4).

**Figure 5:**
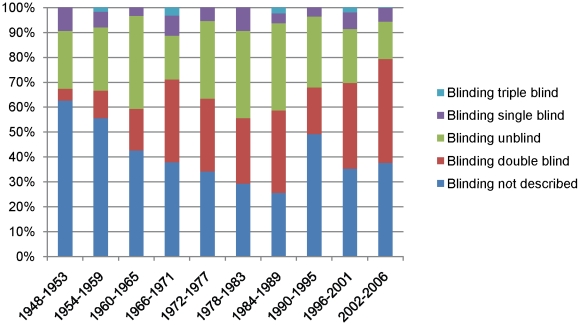
Level of blinding over time from 1948-2006.

The proportion of double-blind studies increased over time (P<0.0001). To assess if the level of blinding changed over time, we ordered the 5 possible responses from least blinding to most blinding (unblind, not described, single, double, triple-blind) and found a significant increase (P = 0.007). As shown in Figure 5, many trials still do not describe their level of blinding; even in the most recent time period, 2002–2006, 37.7% of authors did not include this description.

**Figure 6:**
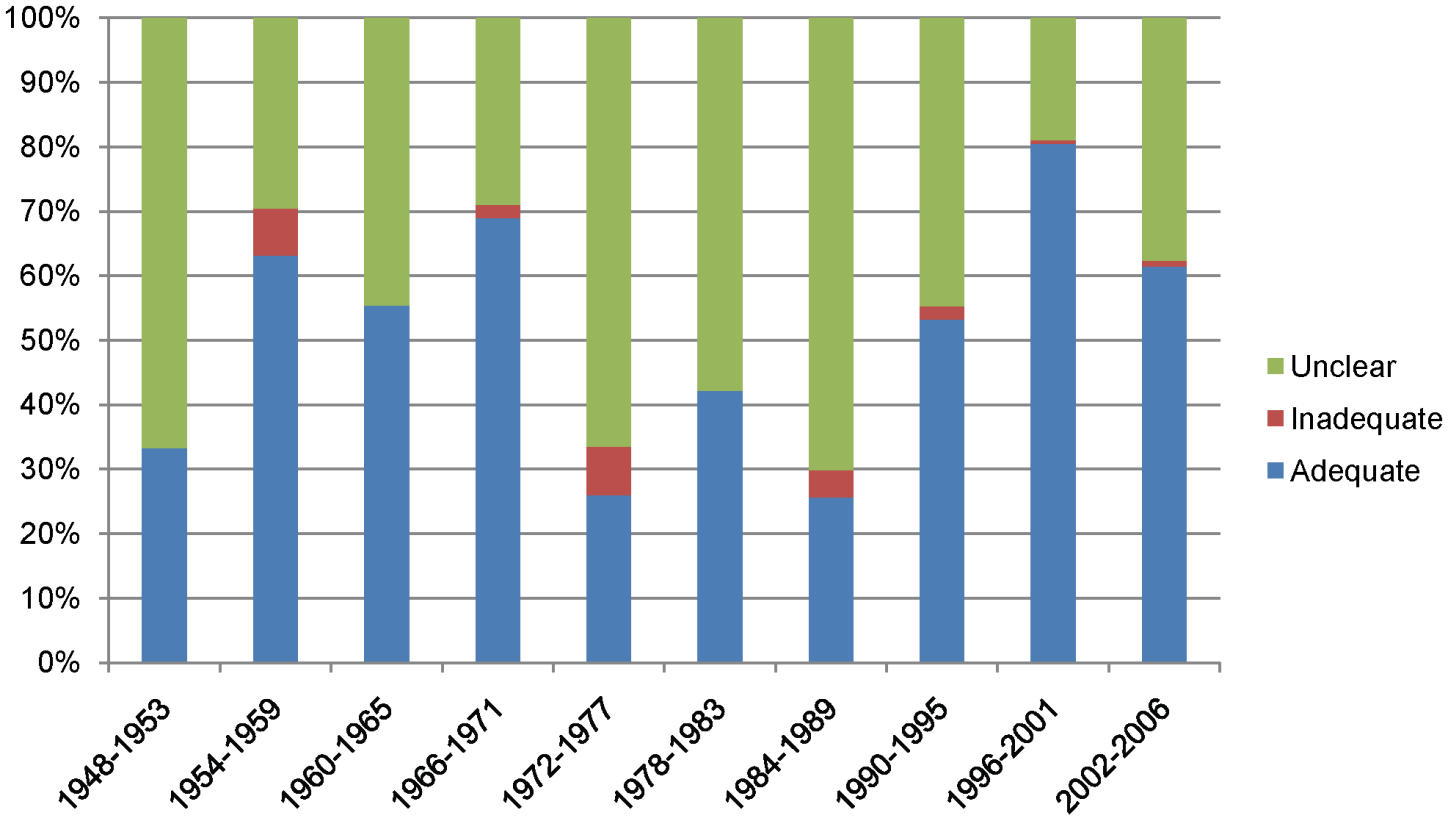
Allocation concealment over time (RCTs only).

To estimate the progression of allocation concealment over time, we ordered the three possibilities as inadequate, unclear, adequate, and found that there was an increase in adequate allocation concealment over time (P<0.0001). However, allocation concealment was inadequately reported in the majority of studies across the entire time period (Figure 6).

Additionally, our regression analysis found an increase in reporting of the following characteristics over time: adequate description of withdrawals and losses to follow-up (p-value<0.0001); sample size calculations (P-value<0.0001); and intention-to-treat analysis (P<0.0001).

#### 3. Relationship between funding and authors' conclusions

For each study, we recorded if the authors declared a source of funding for the study and whether the funding came from industry (pharmaceutical or other corporations). The non-pharmaceutical companies in our sample all had interests related to the topic of the study. For example, M & R Laboratories and its successor company Ross Laboratories, manufacturer of infant formula, supported several studies involving infant formula. The IVAC Corporation, a manufacturer of infant incubators, supplied equipment for one study. IBM provided five programmed instruction machines for children with learning disabilities for a study testing the effectiveness of these machines.

We analysed the relationship between declared funding from industry and whether the overall conclusions of the article were positive or negative (Table 3). In order to decide whether the overall study conclusion was positive, negative, or unclear, reviewers considered the objective or aim of that study. If the authors of the study stated that they had achieved their objective or aim, the overall conclusions were considered positive. If the authors stated they did not achieve the objective or aim, overall conclusions were reported as negative. A decision of “unclear” was made if the authors did not clearly state their objective or aim, or if the conclusions to their study were inconclusive. The probability of reporting positive conclusions was significantly higher for studies with industry funding (p = 0.028) (Table 4). There is evidence that the proportion of studies with industry funding decreased slightly over time (P = 0.003).

**Table 3:** Authors' conclusions by funding source.

Industry Funding	Conclusions	Estimate (95% CI)
Yes	Positive	86.0% (76.0%, 96.1%)
	Negative	9.6% (0.4%, 18.8%)
	Unclear	4.4% (0.0%, 8.9%)
No	Positive	69.1% (58.0%, 80.3%)
	Negative	21.6% (11.5%, 31.7%)
	Unclear	9.3% (2.3%, 16.2%)

p-value comparing probability of positive conclusion: 0.028.

p-value comparing probability of negative conclusion: 0.085.

**Table 4:** Industry funding over time.

	1948–1953	1954–1959	1960–1965	1966–1971	1972–1977	1978–1983	1984–1989	1990–1995	1996–2001	2002–2006
Yes	18.20%	25.00%	13.20%	25.00%	13.50%	9.30%	10.00%	5.20%	17.50%	12.30%
No	11.40%	15.60%	31.90%	29.50%	33.80%	25.00%	24.20%	29.80%	27.90%	39.70%
Unclear	70.50%	59.40%	54.90%	45.40%	52.70%	65.70%	65.80%	65.10%	54.60%	48.00%

## Discussion

The size, span and comprehensiveness of our register have allowed us to describe the state of pediatric research over a period of almost sixty years. This is the first comprehensive mapping of research in pediatrics in this way. The results highlight gaps and inadequacies in the research conducted to date and provide a baseline to examine future developments in pediatric research.

Our map of this research shows several encouraging trends, although all of these developments come with some caveats. For example, we found that the number of clinical trials published each year has steadily increased. This is possibly a positive development assuming that increasing number of trials have led to greater amount of knowledge about the effects of treatment. This result is offset by the fact that it is clear that, as shown in Figure 1, the rate of publication of controlled trials in pediatrics has not increased at the same rate as for trials as a whole. It is also encouraging that the methodological quality, as indicated by Jadad scores along with several other quality indicators, increased over time. Further, the proportion of trials that were randomized also increased; this proportion was calculated with the denominator being all studies, while the numerator was the number described as randomized.

However, our results demonstrate that it is starkly evident that there is room for considerable future progress. The median Jadad score did not exceed 2 out of a maximum of 5 points in any particular time period. Allocation concealment, although generally improving over time, was still classed as inadequate or unclear in 83% of trials in our most recent time period, and in no time period was it classed as adequate in more than 26.5% of cases (1996–2001). This highlights the issue of inadequate reporting, as does the fact that in over the time periods 25.6% to 62.8% of authors did not report on their blinding techniques. Of course, a limitation of any quality assessment endeavour is that study authors do not necessarily report on all methodological aspects of their trial. Trial authors might not report on certain methodological aspects, even though these may have been done to a high standard in the trial. This fact might be especially important in investigating historical trends, where increasing recognition over time of the need to report features such as allocation concealment might lead to apparent improvements in trial quality, simply because journal editors are requiring that authors be more descriptive about their methods.

Recent work has demonstrated that published studies involving adults are much more likely than those involving children to be RCTs or systematic reviews, and that studies of therapies constitute a lower percentage of overall published trials in children than in adults[20]. Our work amplifies this concern by showing that, even when a high-quality study design is used in pediatric research, the conduct and reporting of the trial is often inadequate. Driven by concern about the potential impact of this poor reporting on the quality and applicability of child health trials, a group of pediatric clinicians, methodologists and policy makers, including some of the authors of this article, have formed the StaR Child Health initiative, which is aimed at promoting the use of evidence-based standards or guidance for clinical studies with children [21]. These standards will be developed in several domains, building on recent work by StaR Child Health members in areas such as core outcome measures and risk of bias assessment[22], [23].

Our results are congruent with studies of methodological quality in other disciplines. A recent systematic review examining 35 studies of methodological quality and trends in various medical disciplines [24] found that progress over time in these disciplines in key variables such as allocation concealment, blinding and randomization was variable. For example, they report that, among the studies included in their review, the proportions of RCT authors reporting an adequate method of allocation concealment did not ever exceed 50%, and in several studies, were less than 20%.

The increase in the number of multi-centre and multinational trials is also encouraging. This trend may be a response to the difficulty of designing adequately-powered trials of interventions used to treat pediatric conditions which are frequently comparatively rare.

We noted several points which warrant further research. For example, we are unable to ascertain the cause of the dramatic jump in the number of trials, noted in 2002 and subsequently. One possible explanation is the impact of previously mentioned legislation in the US and subsequently the EU that brought in more tangible incentives and repercussions for drug manufacturers with regard to testing their products on children. The goal of this legislation was to encourage pediatric study; however, it has been shown that pediatric exclusivity provisions can generate lucrative returns on investment [25], [26], [27]. It would also be interesting to see if this legislation is related at all to the trend we noted of a decrease in child-only studies over time, as manufacturers may have moved to including children in larger studies in order to prolong market exclusivity of drugs with both pediatric and adult indications but proportionately a much larger adult market share.

It would also be interesting to analyse changes in the number of trials currently being conducted and/or registered, to compare these figures with the number of trials published. Recent work analysing the rate of registration of pediatric trials suggests that authors of pediatric trials have been slow to prospectively register their studies, despite the fact that many journals stipulate that prospective registration is a prerequisite for publication[28], [29]. Therefore it seems likely that analysing the number of registered trials would substantially underestimate the number of trials actually being conducted.

Our findings that trials supported by industry funding tend to have positive conclusions more often than those reporting other sources of funding are similar to what has been demonstrated in many other medical disciplines. In a qualitative systematic review of 19 studies examining the relationship of pharmaceutical industry funding and clinical trial results, Sismondo [30] demonstrated a broad range of situations in which industry funding has effects on trial results, including over-publication of positive results and under-publication of negative ones, design biases, duplication of known positive results, and influences on the interpretation of data. He argues that no further research is needed in this area, since the existing work is so conclusive.

To date, little work has been done to examine this relationship in pediatric research. Nkansah et al [31] conducted a small study examining the relationship between industry sponsorship and study outcomes in randomized trials assessing calcium supplementation in healthy children. They did not find a significant association; however, the studies in their sample showed minimal variability in study results or sponsorship (16/19 studies were industry funded to some degree, and 17/19 reported statistically significant improvement of bone density with calcium supplementation), which limited the authors' ability to detect associations. Also, as our sample indicated that the proportion of trials reporting industry funding had decreased slightly over time, it would be interesting to examine this trend in more detail.

### Study limitations

The sampling procedure used for our trials was a necessity, given the size of the database we were working with. However, it did create a lack of precision in certain aspects of our analysis. For example, although we were able to find trends over time in quality indicators, we could not identify certain key years or time periods in which the quality increased more than others. This meant that we could not, for example, establish whether the 1996 publication of CONSORT led to increases in the quality of pediatric trials{Kane, 2007 400071/id;Moher, 2001 400072/id;Plint, 2006 400073/id}. Future research to examine the impact of CONSORT on pediatric trials would be of value. At the time of the random sampling we had not yet translated 21 non-English studies that were later eligible for inclusion. We were able to describe trends in pediatric trials as a whole; however, further work in specific areas of pediatric health would illuminate strengths and weaknesses of study design and reporting in different disciplines.

Another source of limitations comes from our reliance on CENTRAL as a source for our Trials Register and hence for the sample for this study. The fact that we did not carry out a search for grey literature introduces the possibility of publication bias. As well, CENTRAL is not a comprehensive source of all published trials. For example, Arabic, Chinese and other character-based languages are not supported by the current software. It is also possible that the way CENTRAL has been created has led to artefacts in relation to apparent trends in its content; for example, handsearching activity by Cochrane Review Groups may favour more recent trials.

It was also not possible with this study design to directly determine the impact of increasing numbers of trials on child health outcomes.

## Materials and Methods

The Cochrane Collaboration exists to improve health care by producing and disseminating systematic reviews of evidence about health care interventions. Within the Collaboration, groups known as “Fields” facilitate the production and dissemination of reviews related to their specialist area of health care. The Cochrane Child Health Field (the Field) carries out these responsibilities for the child health community.

The Field maintains a reference-based Trials Register that contains approximately 30,000 bibliographic records of child health randomised controlled trials (RCTs) and controlled clinical trials (CCTs), published from 1948 onwards. Records within the Trials Register originate from the Cochrane Central Register of Controlled Trials (CENTRAL). CENTRAL is comprised of records of studies from Medline, Embase, handsearch results, grey literature and trials registers of 52 Cochrane Review Groups that are published internationally in many languages[32]. CENTRAL has also been demonstrated to be the single best source of controlled trials for Cochrane reviews[33]. To build the Child Health Field Trials Register, we searched CENTRAL in July 2002; an update was conducted in March 2006. A sensitive pediatric search filter was used for these searches, which is reproduced in Table S2. Included studies were identified by scanning the title and abstract of each record. If the reviewer could not determine eligibility from the title and abstract, the full text of the study was retrieved.

The inclusion criteria for the Trial Register are as follows:

- Study design must be an RCT or CCT, as defined by the Cochrane Collaboration.[19]
- All pregnancy studies are excluded. The lower age limit of the register will be the moment after birth.
- Studies on breastfeeding are included because breastfeeding is a form of infant nutrition.
- If the lower age limit is between 13 and 18 and the upper age limit is 22+ years, the study is excluded.
- If the lower age limit is between 13 and 17 years and the upper age limit is 21 years or less, the study is included[18].

This register is a unique resource for the systematic evaluation of the development of randomized controlled trials and controlled clinical trials across the discipline of pediatrics. These two types of study designs are considered to yield the most reliable information on the efficacy and effectiveness of healthcare interventions.

### Comparison of pediatric trials to total number of trials

In March, 2008, we searched the Cochrane Central Register of Controlled Trials (CENTRAL), for the number of trials for each year between 1948 and 2005. This was done year by year by restricting the date range to an individual year. The results were then compared to the number of trials in the Field Trials Register, to establish the percentage of trials for each year that were in the area of child health. We did not include 2006 in this analysis because the search update for the Trials Register was conducted in March 2006 and therefore our numerator would have been inaccurate.

### Sample identification

Initially, we started with a sample of 588 trials, published between 1948 and 2006. All studies in the Trials Register that were published prior to 1960 were automatically included because of the small number of studies in these years (n = 118). Trials from all the years from 1960 to 2006 were sampled (using simple random sampling) at 10 trials per year. During data extraction studies that were found not be RCTs or CCTs - or that did not meet the age range criteria of the Trials Register - were excluded from our sample and were also removed from the Trials Register. We replaced the excluded studies by randomly selecting another study when there were other studies published during the same years. However, for the years 1948 to 1959, there were no other trials to draw from, so when trials from these years were excluded they could not be replaced; therefore, the final number of trials from this time period was 108. Hence we had a final total of 578 trials available for analysis. We did not limit inclusion of trials in our sample by language of publication. Table S1 presents the percentage of trials from each year that were in our sample.

Our sample size was dictated by the time and resources our centre possessed to perform the data extraction. The decision to utilize a stratified random sampling with an equal number of trials represented from each year was to obtain equal representation from each time period so that changes over the years could be evaluated. While this sampling allocation method may decrease the accuracy of estimates on the global level, it will increase our estimates for specific domains—particularly the early years when number of trials was scarce. While this gives us relatively small sampling fractions in later years compared to earlier years, it is important to note that sampling fraction is a very small part of the variance of computed estimates.

### Data extraction

Data were extracted using a structured electronic form and covered the following categories: publication characteristics (e.g., year of publication, journal, country of corresponding author); trial characteristics (e.g. nature of intervention, placebo-controlled, diagnostic category); and overall conclusions (did the study report statistical significance for at least one outcome (yes/no), if yes, what did it favour, overall authors' conclusions); and methodological quality assessment.

Five staff members carried out the data extraction. Two of these did the majority (72.4% of the studies). Staff was able to extract data from studies in French, German, Spanish, Portuguese, Polish, Italian and Chinese. Studies in Japanese, Dutch, Swedish and Slovenian were extracted by volunteers under the guidance of staff. We did not exclude any studies based on language.

Studies for which the staff member doing the data extraction had any questions were double-checked by the centre's scientific director, and questions were resolved by consensus. A random sample of 10% of the studies were pulled and double-checked by a staff member who had not done any of the data extraction. Differences were resolved by consensus.

### Assessment of Methodological Quality

All studies were scored for methodological quality using the Jadad scale[20], with additional questions to describe allocation concealment, blinding, and whether an intention-to-treat analysis was described.

### Division into time periods

To analyse trends over time, we divided our fifty-eight-year sample into nine six-year time periods and one five-year period (2002–2006). This division created a sufficient sample to perform the statistical analysis. For the six-year time periods, a sample of 60 studies would give us estimates to within plus or minus 13% of proportions (regardless of the sampling proportion).

### Statistical Analysis

Weighted Horvitz-Thompson estimators were used in all computations to account for the stratified sampling design. Descriptive statistics were presented as either means with 95% confidence intervals for continuous variables (e.g. Jadad score) or as percentages with 95% confidence intervals for dichotomous and categorical data. Weighted regression analysis, both linear and logistic was performed using the same Horvitz-Thompson weights. Trends over time were computed on the base years, not using the summary time periods described above. All computations were performed using SAS 9.1.

## Supporting Information

Table S1 Percentage of trials from each year in sample.
(0.01 MB DOCX)

Table S2 Search filter for CENTRAL.
(0.01 MB DOCX)
