# Supplementary material for: Controlled Trials in Children: Quantity, Methodological Quality and Descriptive Characteristics of Pediatric Controlled Trials Published 1948-2006
Source: PLoS One. 2010 Sep 30;5(9):e13106. doi: 10.1371/journal.pone.0013106 (PMC2948021; doi:10.1371/journal.pone.0013106)
Supplement: Table S1 — Percentage of trials from each year in sample. (0.01 MB DOCX) [file pone.0013106.s001.docx]

Table 5. Percentage of Trials From Each Year in Sample

| Year | Estimated number of trials | Proportion |
| --- | --- | --- |
| 1948 | 3 | 100.00% |
| 1949 | 5 | 100.00% |
| 1950 | 3 | 100.00% |
| 1951 | 6 | 100.00% |
| 1952 | 18 | 100.00% |
| 1953 | 14 | 100.00% |
| 1954 | 5 | 100.00% |
| 1955 | 10 | 100.00% |
| 1956 | 16 | 100.00% |
| 1957 | 8 | 100.00% |
| 1958 | 15 | 100.00% |
| 1959 | 15 | 100.00% |
| 1960 | 29 | 34.48% |
| 1961 | 26 | 38.46% |
| 1962 | 16 | 62.50% |
| 1963 | 32 | 31.43% |
| 1964 | 29 | 34.48% |
| 1965 | 33 | 30.30% |
| 1966 | 64 | 15.71% |
| 1967 | 66 | 15.15% |
| 1968 | 80 | 12.50% |
| 1969 | 73 | 13.79% |
| 1970 | 74 | 13.48% |
| 1971 | 95 | 10.58% |
| 1972 | 109 | 9.17% |
| 1973 | 136 | 7.35% |
| 1974 | 175 | 5.71% |
| 1975 | 214 | 4.67% |
| 1976 | 176 | 5.68% |
| 1977 | 211 | 4.74% |
| 1978 | 273 | 3.67% |
| 1979 | 278 | 3.59% |
| 1980 | 313 | 3.52% |
| 1981 | 343 | 2.92% |
| 1982 | 385 | 2.60% |
| 1983 | 508 | 1.97% |
| 1984 | 418 | 2.40% |
| 1985 | 569 | 1.76% |
| 1986 | 613 | 1.63% |
| 1987 | 648 | 1.54% |
| 1988 | 607 | 1.65% |
| 1989 | 814 | 1.23% |
| 1990 | 935 | 1.07% |
| 1991 | 747 | 1.34% |
| 1992 | 707 | 1.41% |
| 1993 | 935 | 1.07% |
| 1994 | 883 | 1.13% |
| 1995 | 1017 | 0.98% |
| 1996 | 884 | 1.13% |
| 1997 | 1031 | 0.97% |
| 1998 | 1150 | 0.87% |
| 1999 | 972 | 1.03% |
| 2000 | 1093 | 0.91% |
| 2001 | 538 | 1.86% |
| 2002 | 2329 | 0.43% |
| 2003 | 1971 | 0.51% |
| 2004 | 1944 | 0.51% |
| 2005 | 1825 | 0.55% |
| 2006 | 21 | 47.62% |
