# Supplementary material for: Controlled Trials in Children: Quantity, Methodological Quality and Descriptive Characteristics of Pediatric Controlled Trials Published 1948-2006
Source: PLoS One. 2010 Sep 30;5(9):e13106. doi: 10.1371/journal.pone.0013106 (PMC2948021; doi:10.1371/journal.pone.0013106)
Supplement: Table S2 — Search filter for CENTRAL. (0.01 MB DOCX) [file pone.0013106.s002.docx]

Table S2 – Search Filter for CENTRAL

1. CHILD*
2. INFAN*
3. ADOLESCEN*
4. NEWBORN*
5. PRESCHOOL*
6. KINDERGARTEN*
7. NURSERY SCHOOL
8. ELEMENTARY SCHOOL
9. TEEN or TEENS or TEENAGE*
10. UNDERAGE*
11. PREEMIE*
12. NEONAT*
13. YOUTH or YOUTHS
14. UNDERAGE*
15. BABY or BABIES
16. PREPUBESCEN* or PUBESCEN*
17. SCHOOLCHILD*
18. DAYCARE*
19. SCHOOLAGE*
20. BOY* or GIRL*
21. OFFSPRING
22. PAEDIATRIC* or PEDIATRIC*
23. JUVENIL*
24. TODDLER*
25. NURSERY or NURSERIES
26. HIGH SCHOOL* or HIGHSCHOOL*
27. PRIMARY SCHOOL*
28. SECONDARY SCHOOL*

29. #1 OR #2 OR #3 OR #4 OR #5 OR #6 OR #7 OR #8 OR #9 OR #10 #11 OR #12 OR #13 OR #14 OR #15 OR #16 OR #17 OR #18 OR #19 OR #20 or #21 or #22 #OR #23 OR #24 OR #25 OR #26 OR #27 OR #28
30. (ADOLESCENC*:KY and ADULT*:KY)
31. ((CHILD:KY or INFANT:KY) or NEWBORN:KY)
32. (#30 not #31)
33. (#29 not #32)
